# Detection of differentially methylated regions from bisulfite-seq data by hidden Markov models incorporating genome-wide methylation level distributions

**DOI:** 10.1186/1471-2164-16-S12-S3

**Published:** 2015-12-09

**Authors:** Yutaka Saito, Toutai Mituyama

**Affiliations:** 1 Biotechnology Research Institute for Drug Discovery, National Institute of Advanced Industrial Science and Technology (AIST), 2-4-7 Aomi, Koto-ku, 135-0064 Tokyo, Japan; 2 Core Research for Evolutional Science and Technology (CREST), Japan Science and Technology Agency (JST), 4-1-8 Honcho, Kawaguchi, 332-0012 Saitama, Japan

**Keywords:** epigenomics, DNA methylation, differentially methylated region (DMR), bisulfite sequencing, hidden Markov model (HMM), beta mixture

## Abstract

**Background:**

Detection of differential methylation between biological samples is an important task in bisulfite-seq data analysis. Several studies have attempted de novo finding of differentially methylated regions (DMRs) using hidden Markov models (HMMs). However, there is room for improvement in the design of HMMs, especially on emission functions that evaluate the likelihood of differential methylation at each cytosine site.

**Results:**

We describe a new HMM for DMR detection from bisulfite-seq data. Our method utilizes emission functions that combine binomial models for aligned read counts, and beta mixtures for incorporating genome-wide methylation level distributions. We also develop unsupervised learning algorithms to adjust parameters of the beta-binomial models depending on differential methylation types (up, down, and not changed). In experiments on both simulated and real datasets, the new HMM improves DMR detection accuracy compared with HMMs in our previous study. Furthermore, our method achieves better accuracy than other methods using Fisher's exact test and methylation level smoothing.

**Conclusions:**

Our method enables accurate DMR detection from bisulfite-seq data. The implementation of our method is named ComMet, and distributed as a part of Bisulfighter package, which is available at http://epigenome.cbrc.jp/bisulfighter.

## Background

Cytosine methylation is an epigenetic modification that affects many biological processes including normal development and pathogenesis [1]. Genome-wide profiling of cytosine methylation is enabled by bisulfite-seq, where unmethylated cytosines are converted and sequenced as thymines [2]. In bisulfite-seq data analysis, a fundamental task is alignment of bisulfite-converted reads to a reference genome, and thus numerous tools have been already developed [3-6]. On the other hand, methods for downstream tasks after read alignment have been relatively limited [7]. Among them, one of the most important is detection of differential methylation between biological samples [8]. Differential methylation analyses can be divided into two categories: those focusing only on pre-specified regions such as known transcription factor binding sites (*e.g*. [9]), and those for de novo finding of differentially methylated regions (DMRs) as novel candidates of regulatory elements (*e.g*. [10]). In this paper, we address the latter case, which is more challenging due to the necessity for determining exact boundaries of DMRs.

DMR detection has been attempted by two-step procedures: first, differentially methylated cytosines (DMCs) are detected by comparison of alignment results between samples; then, DMCs at neighbor positions are grouped as contiguous DMRs by certain distance criteria. Most studies have focused mainly on the first step, and proposed to detect DMCs using Fisher's exact test [10], Student's t-test with methylation level smoothing [11], and logistic regression test [12]. Additionally, many methods have been developed for detecting DMCs based on a variety of probability models [13-15]. In contrast, there have been much less studies on methods for grouping DMCs into DMRs. Although fixed-length distance criteria (*e.g*. sliding windows) have been conventionally used, such strategies depend on the choice of distance parameters (*e.g*. window sizes). Unfortunately, it is difficult to adjust distance parameters empirically because DMR lengths range from hundreds of base pairs as in CpG islands, to millions of base pairs as in cancer aberrations [16].

To address this problem, we have recently proposed a framework for DMR detection based on hidden Markov models (HMMs) [6]. Unlike the two-step procedures, HMMs can integrate detection and grouping of DMCs as joint probability models using emission and transition functions, respectively. Moreover, HMMs enable us to adjust their parameters by well-established learning algorithms so that they incorporate useful information for DMR detection. In particular, we have observed that DMCs exhibit distance distributions distinct from cytosines whose methylation is not changed. Therefore, we have adjusted parameters of transition functions so that they fit these distance distributions. Thanks to this strategy, our method has improved DMR detection accuracy, especially on determining exact boundaries of DMRs. We note that HMM-based DMR detection has also been employed for methylation data other than bisulfite-seq such as Infinium BeadChip [17] and MBDCap-seq [18].

While our previous study has shown the effectiveness of transition functions in HMM-based DMR detection, there is still room for improvement in the design of emission functions. As mentioned above, many studies have proposed various probability models for detecting DMCs [13-15]. An important suggestion from these studies is that DMC detection at individual cytosine sites can be improved by considering probability distributions of methylation levels collected from all genomic cytosine sites. This implies that the information of genome-wide methylation level distributions may also be useful for DMR detection. However, the probability models in [13-15] are specifically developed for DMC detection, and thus cannot be directly applied to the emission functions for HMM-based DMR detection.

In this paper, we describe new emission functions for HMM-based DMR detection from bisulfite-seq data. We show that the emission functions in our previous study [6] have an empirical parameter to represent methylation levels used in binomial models for aligned read counts. From this viewpoint, we propose new emission functions that replace the empirical parameter by beta mixtures for incorporating genome-wide methylation level distributions. We also develop unsupervised learning algorithms to adjust parameters of the beta-binomial models depending on differential methylation types (up, down, and not changed). In experiments on both simulated and real datasets, the new emission functions improve DMR detection accuracy compared with the old ones. Furthermore, our HMM-based method achieves better accuracy than other methods using Fisher's exact test and methylation level smoothing.

## Methods

In this section, we describe a new method for DMR detection from bisulfite-seq data. The method uses new emission functions with an HMM-based framework called ComMet which we developed in our previous study [6]. We first review ComMet, and show that emission functions in our previous study have an empirical parameter to represent methylation levels used in binomial models for aligned read counts. Then, we design new emission functions replacing this empirical parameter by beta mixtures for incorporating genome-wide methylation level distributions. We also present unsupervised learning algorithms to adjust parameters of the beta-binomial models depending on differential methylation types (up, down, and not changed).

### HMM-based DMR detection from bisulfite-seq data

**Figure 1:**
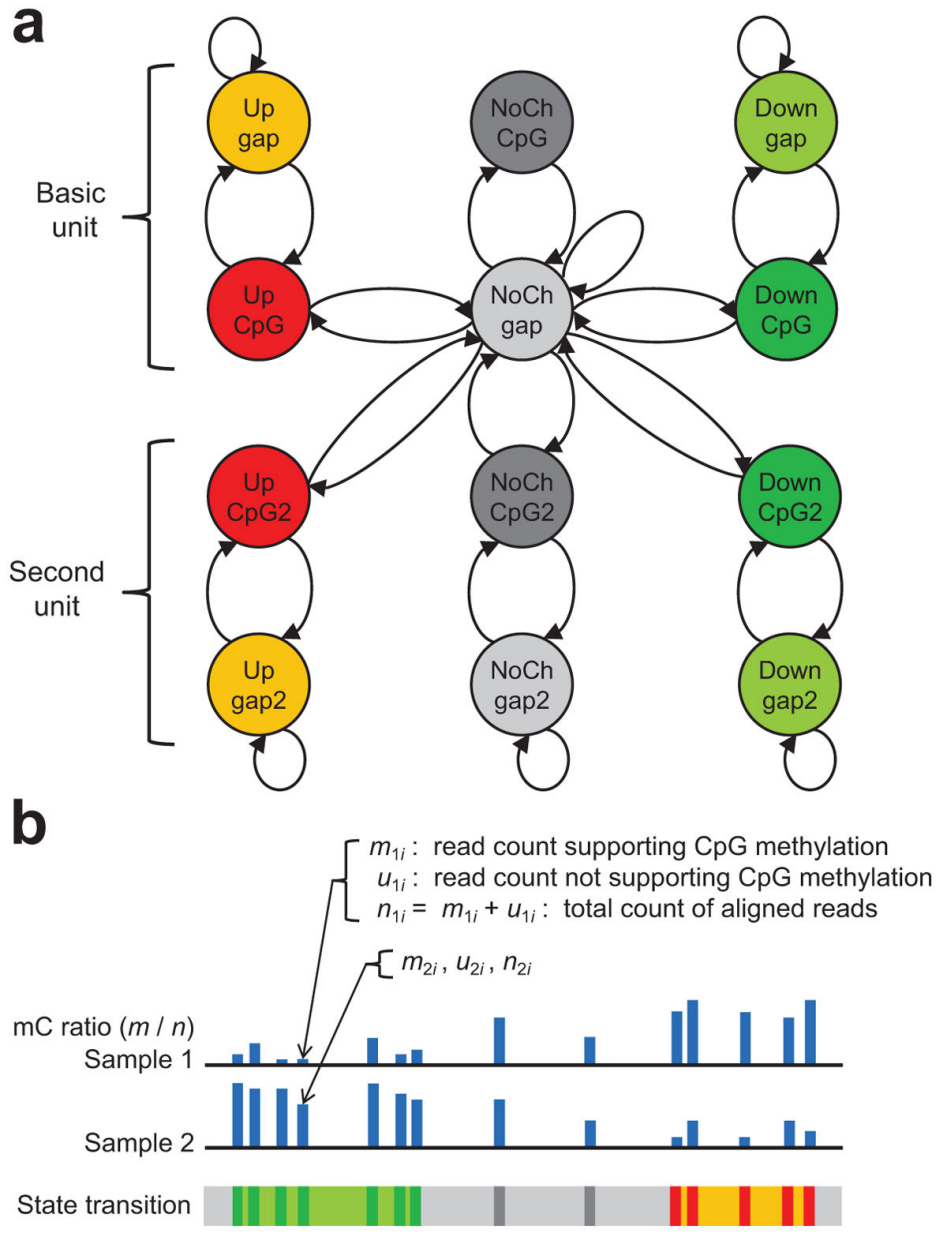
**ComMet: an HMM-based framework for DMR detection from bisulfite-seq data**. (a) HMM architecture. The HMM has pairs of states for CpG positions and their interval positions (named *gap*), each of which has three types of differential methylation: hypermethylation (Up), hypomethylation (Down), and no change (NoCh). Transition probabilities among Up, Down, and NoCh states represent distinct distance distributions among DMCs. Throughout this study, we use the dual architecture consisting of the basic and second units since it can achieve better accuracy than the basic unit only [6]. (b) Example of input bisulfite-seq data and corresponding state transitions. Colors in the state transition track correspond to those in (a).

In our previous study [6], we developed ComMet, an HMM-based framework for DMR detection from bisulfite-seq data (Figure 1). The motivation for employing HMMs came from our observation of real data where DMCs showed distance distributions distinct from CpGs whose methylation was not changed. We incorporated these distributions into transition functions of HMMs. ComMet uses the state transition diagram shown in Figure 1a where transition probabilities among Up, Down, and NoCh states represent distinct distance distributions among DMCs. ComMet adjusts transition probabilities for each dataset to be analyzed using expectation-maximization algorithms. ComMet detects DMRs by dynamic programming algorithms that maximize log-likelihood ratio scores log $\frac{P\left(\text{region},\;dir\right)}{P\left(\text{region},\;\text{NoCh}\right)}$, where *dir *(= Up or Down) is the direction of differential methylation. The output of ComMet is a list of DMRs ranked by their log-likelihood ratio scores.

While transition functions of ComMet incorporated distance distributions of DMCs, the design of emission functions was not well established in our previous study. Given alignment results of bisulfite-converted reads, we can observe the counts of reads supporting CpG methylation as the number of C-C matches, and the counts of reads not supporting CpG methylation as the number of C-T mismatches (Figure 1b). Let us denote the count of reads supporting methylation by *m_si_*, the count of reads not supporting methylation by *u_si_*, and the total count of aligned reads by *n_si_*, for each CpG site *i *and each sample *s *= 1, 2. If a CpG site is differentially methylated, the counts can be considered to be taken from separate probability distributions reflecting the difference of methylation levels between two samples. On the other hand, if a CpG site is not differentially methylated, the counts should be the consequence of the common methylation level. Therefore, in our previous study, emission functions for CpG states were designed as follows:

$$\begin{array}{c} e_{i}^{\text{U}}=\text{Binom}\left(m_{1i}|n_{1i},\;\theta_{1\;i}^{\text{U}}\right)\text{Binom}\left(m_{2\;i}|n_{2\;i},\;\theta_{2\;i}^{\text{U}}\right),\\ e_{i}^{\text{D}}=\text{Binom}\left(m_{1i}|n_{1i},\;\theta_{1i}^{\text{D}}\right)\text{Binom}\left(m_{2i}|n_{2i},\;\theta_{2i}^{\text{D}}\right),\\ e_{i}^{\text{N}}=\text{Binom}\left(m_{1\;i}|n_{1\;i},\;\theta_{0i}^{\text{N}}\right)\text{Binom}\left(m_{2i}|n_{2i},\;\theta_{0i}^{\text{N}}\right), \end{array}$$

where U, D, and N represent Up, Down, and NoCh states, respectively, Binom() is a binomial distribution, and $\theta_{i}^{\cdot }$ is the occurrence probability of reads supporting CpG methylation at the *i*-th CpG site for each differential methylation state. (Note that we use common emission functions between the basic and second units in Figure 1a, and no emission function for gap states.) The problem here is how to model $\theta_{i}^{\cdot }$ depending on differential methylation states. One may consider to use $\theta_{1i}^{\text{U}}=\theta_{1i}^{\text{D}}=m_{1i}/n_{1i}$, $\theta_{2i}^{\text{U}}=\theta_{2i}^{\text{D}}=m_{2i}/n_{2i}$, and $\theta_{0i}^{\text{N}}=\left(m_{1i}+m_{2i}\right)/\left(n_{1i}+n_{2i}\right)$. However, this cannot discriminate the direction of differential methylation due to $\theta_{i}^{\text{U}}=\theta_{i}^{\text{D}}$, and thus is not a suitable choice. In our previous study, we resorted to introduce an empirical parameter *pseudo*, resulting in

$$\begin{array}{c} \theta_{1i}^{\text{U}}=\frac{m_{1i}+pseudo}{n_{1i}+pseudo},\\ \theta_{2i}^{\text{U}}=\frac{m_{2i}}{n_{2i}+pseudo},\\ \theta_{1i}^{\text{D}}=\frac{m_{1i}}{m_{1i}+pseudo},\\ \theta_{2i}^{\text{D}}=\frac{m_{2i}+pseudo}{n_{2i}+pseudo},\\ \theta_{0i}^{\text{N}}=\frac{m_{1i}+m_{2i}}{n_{1i}+n_{2i}}, \end{array}$$

We note that *pseudo *can be regarded as a pseudocount added to actual read counts, playing a role to represent state-dependent methylation levels. For example, if a CpG site has the differential methylation state of Up, we expect that the methylation level is high in the sample 1 and low in the sample 2 (Figure 1b). Accordingly, *pseudo *is added to *m*_1*i *_(supporting CpG methylation in the sample 1) and to *u*_2*i *_(not supporting CpG methylation in the sample 2).

Although the empirical parameter partially solved the problem of designing emission functions, our previous study did not address how to adjust it. The optimal value of *pseudo *depends on the magnitude of read counts *m *and *n *(i.e. sequencing depth). Moreover, it may also depend on underlying biological processes between samples such as normal development and pathogenesis. In fact, as will be shown in the "Results and discussion" section, ComMet with the above emission functions may result in poor accuracy of DMR detection depending on the value of *pseudo*.

### New emission functions and learning algorithms

To design new emission functions for ComMet, we recall that the empirical parameter in our previous study had a role to represent state-dependent methylation levels. This viewpoint leads to the idea that the empirical parameters can be replaced by utilizing genome-wide methylation level distributions observed from real data. To formulate this intuition, we propose new emission functions in the following form:

(1) $$e_{i}^{\text{U}}=\int_{0}^{1}\int_{0}^{1}\text{Binom}\left(m_{1i}|n_{1i},\;\theta_{1}\right)\text{Binom}\left(m_{2i}|n_{2i},\;\theta_{2}\right)p\left(\theta_{1},\;\theta_{2}|\text{U}\right)d\theta_{1}d\theta_{2},$$

(2) $$e_{i}^{\text{D}}=\int_{0}^{1}\int_{0}^{1}\text{Binom}\left(m_{1i}|n_{1i},\;\theta_{1}\right)\text{Binom}\left(m_{2i}|n_{2i},\;\theta_{2}\right)p\left(\theta_{1},\;\theta_{2}|\text{D}\right)d\theta_{1}d\theta_{2},$$

(3) $$e_{i}^{\text{N}}=\int_{0}^{1}\text{Binom}\left(m_{1i}|n_{1i},\;\theta_{0}\right)\text{Binom}\left(m_{2i}|n_{2i},\;\theta_{0}\right)p\left(\theta_{0}|\text{N}\right)d\theta_{0}.$$

Note that new emission functions use probability distributions of state-dependent methylation levels *p*(*θ|·*). This is in contrast to the emission functions in our previous study using fixed values $\theta_{i}^{\cdot }$.

**Figure 2:**
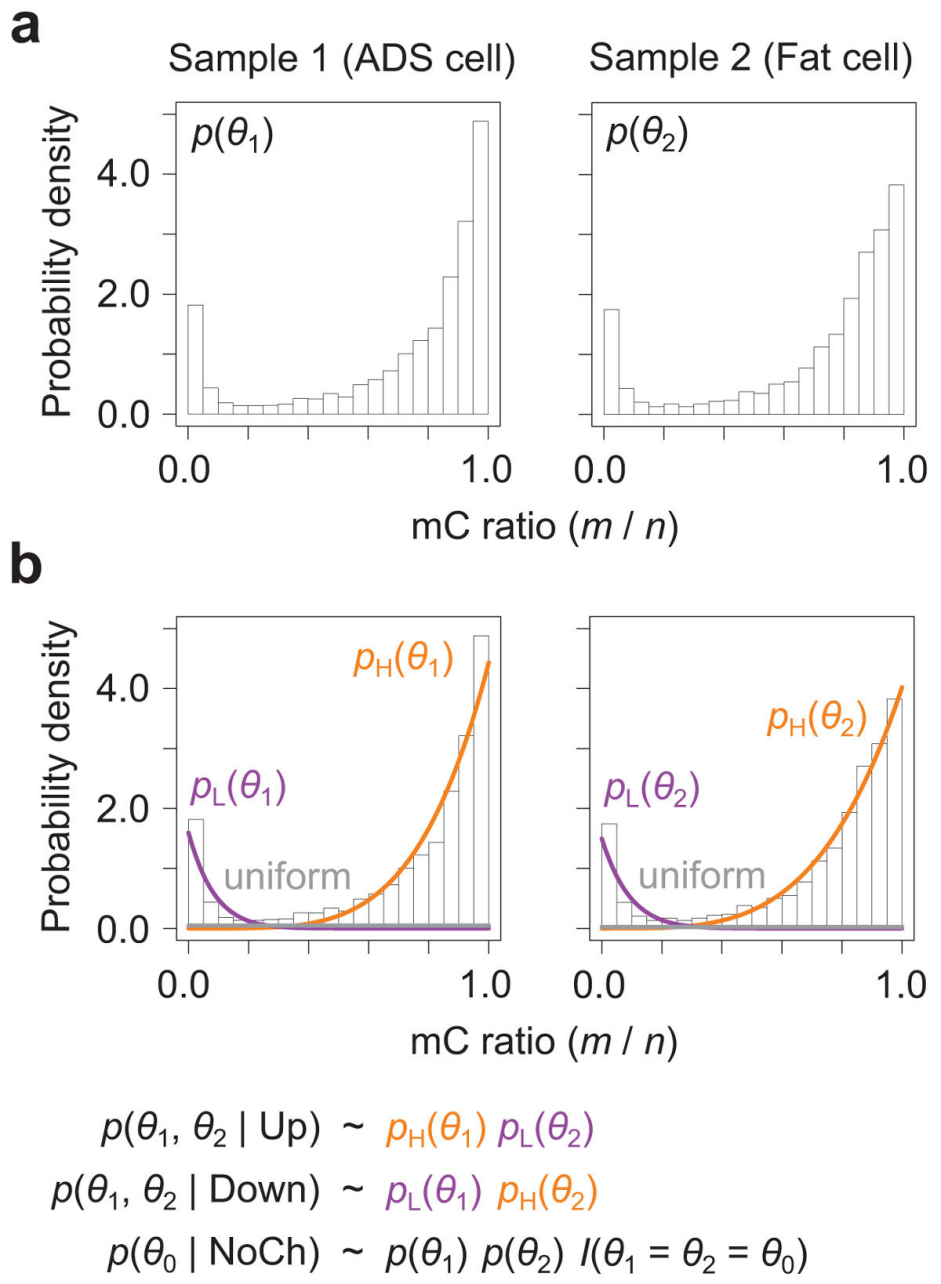
**Learning procedures for new emission functions**. (a) Bimodality of genome-wide methylation level distributions. Real bisulfite-seq data from [10] are shown. (b) Learning parameters for beta mixtures. Genome-wide methylation level distributions are fitted by beta mixtures, and differential methylation states are represented as alterations between two mixture components. The histograms are depicted using all genomic CpG sites, while the probability distributions are estimated using 10000 randomly selected CpG sites.

Next, we present unsupervised learning algorithms for estimating these distributions for each dataset to be analyzed. Figure 2 shows the overview of the algorithms. As shown in Figure 2a, we exploit that methylation levels *m/n *collected from all genomic CpG sites form a distribution with two modes of high and low methylation. Such bimodal methylation level distributions are a common feature observed in many real datasets, and have also been reported by other researchers (*e.g*. 9, 10, 16, 19). Moreover, recent studies have suggested that detection of DMCs at individual cytosine sites can be improved by considering genome-wide methylation level distributions [13-15]. We propose to utilize this information for HMM-based DMR detection. We model genome-wide methylation level distributions by using beta mixtures as follows:

$$\begin{array}{c} p\left(\theta_{1}\right)=p_{\text{H}}\left(\theta_{1}\right)+p_{\text{L}}\left(\theta_{1}\right)+\text{uniform}\\ =w_{\text{H}1}\text{Beta}\left(\theta_{1}|\alpha_{\text{H}1},\;\beta_{\text{H}1}\right)+w_{\text{L}1}\text{Beta}\left(\theta_{1}|\alpha_{\text{L}1},\;\beta_{L1}\right)+w_{\text{unif}1},\\ p\left(\theta_{2}\right)=p_{\text{H}}\left(\theta_{2}\right)+p_{\text{L}}\left(\theta_{2}\right)+\text{uniform}\\ =w_{\text{H}2}\text{Beta}\left(\theta_{2}|\alpha_{\text{H}2},\;\beta_{\text{H}2}\right)+w_{\text{L}2}\text{Beta}\left(\theta_{2}|\alpha_{\text{L}2},\;\beta_{L2}\right)+w_{\text{unif}2}, \end{array}$$

where H and L represent two modes of high and low methylation, each of which is modeled by a beta distribution, and unif represents a background ground methylation level modeled by an uniform distribution (Figure 2b). Using these component distributions, we represent probability distributions of state-dependent methylation levels as follows:

(4) $$p\left(\theta_{1},\;\theta_{2}|\text{U}\right)=\text{Beta}\left(\theta_{1}|\alpha_{\text{H}1},\beta_{\text{H}1}\right)\text{Beta}\left(\theta_{2}|\alpha_{\text{L}2},\;\beta_{\text{L}2}\right),$$

(5) $$p\left(\theta_{1},\;\theta_{2}|\text{D}\right)=\text{Beta}\left(\theta_{1}|\alpha_{\text{L}1},\beta_{\text{L}1}\right)\text{Beta}\left(\theta_{2}|\alpha_{\text{H}2},\;\beta_{\text{H}2}\right),$$

(6) $$\begin{array}{c} p\left(\theta_{0}|\text{N}\right)=\left(w_{\text{H}1}\text{Beta}\left(\theta_{1}|\alpha_{\text{H}1},\beta_{\text{H}1}\right)+w_{L1}\text{Beta}\left(\theta_{1}|\alpha_{\text{L}1},\;\beta_{\text{L}1}\right)+w_{\text{unif}1}\right)\\ \;\;\;\;\;\;\left(w_{\text{H}2}\text{Beta}\left(\theta_{2}|\alpha_{\text{H}2},\beta_{\text{H}2}\right)+w_{L2}\text{Beta}\left(\theta_{2}|\alpha_{\text{L}2},\;\beta_{\text{L}2}\right)+w_{\text{unif}2}\right)\\ \;\;\;\;\;\;I\left(\theta_{1}=\theta_{2}=\theta_{0}\right), \end{array}$$

where *I*() is an indicator function that takes 1 or 0 depending on whether the condition is true or false. This corresponds to represent differential methylation states as alterations between two modes. For example, Up state *p*(*θ*_1_, *θ*_2_|U) is represented as high methylation in the sample 1, *p*_H_(*θ*_1_), and low methylation in the sample 2, *p*_L_(*θ*_2_). By substituting the equations 4-6 into the equations 1-3, the new emission functions are finally written as

(7) $$e_{i}^{\text{U}}=\left(\begin{array}{c} n_{1i}\\ m_{1i} \end{array}\right)\left(\begin{array}{c} n_{2i}\\ m_{2i} \end{array}\right)\frac{B\left(m_{1i}+\alpha_{\text{H}1},\;u_{1i}+\beta_{\text{H}1}\right)}{B\left(\alpha_{\text{H}1},\;\beta_{\text{H}1}\right)}\frac{B\left(m_{2i}+\alpha_{\text{L}2},\;u_{2i}+\beta_{\text{L}2}\right)}{B\left(\alpha_{\text{L}2},\;\beta_{\text{L}2}\right)},$$

(8) $$e_{i}^{\text{D}}=\left(\begin{array}{c} n_{1i}\\ m_{1i} \end{array}\right)\left(\begin{array}{c} n_{2i}\\ m_{2i} \end{array}\right)\frac{B\left(m_{1i}+\alpha_{\text{L}1},\;u_{1i}+\beta_{\text{L}1}\right)}{B\left(\alpha_{\text{L}1},\;\beta_{\text{L}1}\right)}\frac{B\left(m_{2i}+\alpha_{\text{H}2},\;u_{2i}+\beta_{\text{H}2}\right)}{B\left(\alpha_{\text{H}2},\;\beta_{\text{H}2}\right)},$$

(9) $$\begin{array}{c} e_{i}^{\text{N}}=\frac{1}{Z}\left(\begin{array}{c} n_{1i}\\ m_{1i} \end{array}\right)\left(\begin{array}{c} n_{2i}\\ m_{2i} \end{array}\right)\underset{s\in \left\{\text{H}1,\;\text{L}1,\;\text{unif}1\right\}}{\sum }\underset{t\in \left\{\text{H}2,\;\text{L}2,\;\text{unif}2\right\}}{\sum }w_{s}w_{t}\\ \;\;\frac{B\left(m_{1i}+m_{2i}+\alpha_{s}+\;\alpha_{t}-1,\;u_{1i}+u_{2i}+\beta_{s}+\beta_{t}-1\right)}{B\left(\alpha_{s},\;\beta_{s}\right)B\left(\alpha_{t},\;\beta_{t}\right)}, \end{array}$$

(10) $$\begin{array}{c} Z=\underset{s\in \left\{\text{H}1,\;\text{L}1,\;\text{unif}1\right\}}{\sum }\underset{t\in \left\{\text{H}2,\;\text{L}2,\;\text{unif}2\right\}}{\sum }w_{s}w_{t}\\ \;\;\frac{B\left(\alpha_{s}+\alpha_{t}-1,\;\beta_{s}+\beta_{t}-1\right)}{B\left(\alpha_{s},\;\beta_{s}\right)B\left(\alpha_{t},\;\beta_{t}\right)}, \end{array}$$

where *B*() is a beta function, and *α*_unif1 _= *β*_unif1 _= *α*_unif2 _= *β*_unif2 _= 1 by definition of uniform distribution.

The parameter estimation of *w*., *α*., and *β*. involves several technical issues. First, we perform maximum likelihood estimation that maximizes the likelihood of read counts *m *and *n*, rather than methylation levels *m/n*. Read counts preserve the information of sequencing depth (i.e. the magnitude of read counts), which is cancelled in methylation levels. Therefore, this enables to incorporate the information of sequencing depth into parameter values, thereby to overcome the drawback of the previous emission functions where the optimal value of *pseudo *depends on sequencing depth. The estimation problem is regarded as maximum likelihood estimation for beta-binomial mixtures, and thus can be solved as a simpler case of well-established maximum likelihood estimation for Dirichlet-multinomial mixtures described in [20]. Second, we can reduce the computational cost by only using read counts from a small number of randomly selected CpG sites. As shown in Figure 2, the histograms depicted using all genomic CpG sites are well fitted by the probability distributions estimated from 10000 CpG sites. Thus, we use 10000 CpG sites also for other datasets throughout this study. Third, we need to restrict the ranges of parameter values so that the integral in the equation 3 is tractable, and each beta component distribution corresponds to exactly one mode of methylation levels. Accordingly, parameter estimation is performed under the constraints of *α*., *β*. ≥ 1 and *β*_H1 _= *β*_H2 _= *α*_L1 _= *α*_L2 _= 1.

## Results and discussion

To evaluate DMR detection accuracy, we conducted experiments on both simulated and real datasets. Unfortunately, there is no database of gold standards for benchmarking DMR detection (*i.e*. true biological DMRs). Therefore, we employ multilateral evaluation using a series of simulated and real datasets. The overall protocols are similar to those used in [6]. In experiments on simulated data, detected DMRs were evaluated for their overlap with simulated true DMRs. In experiments on real data, detected DMRs were evaluated for agreement with gene expression and DNase I hypersensitivity.

We compared DMR detection accuracy between ComMet using the new emission functions and that using the old ones. In addition, we also compared new ComMet with other methods using Fisher's exact test [10] and methylation level smoothing [11]. We used LAST [5] to align bisulfite-converted reads to reference sequences. The alignment results were used as the common input for each DMR detection method.

### Experiments on simulated data

We simulated bisulfite-converted reads using DNemulator [5]. The human chromosome × (chrX) was used as a reference. Methylation levels were assigned for all CpG sites in the chrX. 87-bp sinlgle-end reads were generated from random loci in the chrX with cytosines converted to thymines according to their methylation levels. Quality values were attached to reads according to SRR094461 in the Sequence Read Archive (http://www.ncbi.nlm.nih.gov/sra), which is bisulfite-seq data produced by the Illumina's platform. These reads were treated as the dataset for the sample 1. Next, 100 random regions were defined as DMRs for Up or Down, and methylation levels of all CpG sites in these regions were changed to the maximum or the minimum, respectively. Reads were again generated, and treated as the dataset for the sample 2. To test the effects of sequencing depth, we varied the number of generated reads for each dataset from 1 to 50 million (M). We also varied the length of simulated DMRs by preparing four versions of datasets: 50 bp, 500 bp, 5 kbp, and 50 kbp.

We evaluated DMR detection accuracy using the rate of correct predictions in the top 100 DMRs detected by each method. A correct prediction was defined as a simulated true DMR reciprocally overlapped with a detected DMR in a certain proportion of their lengths. For example, a correct prediction with 50% reciprocal overlap was counted only if the length of the overlapping region was larger than half the length of the simulated true DMR, and half the length of the detected DMR. Similarly, we also defined correct predictions for 90% and 99% reciprocal overlaps.

**Figure 3:**
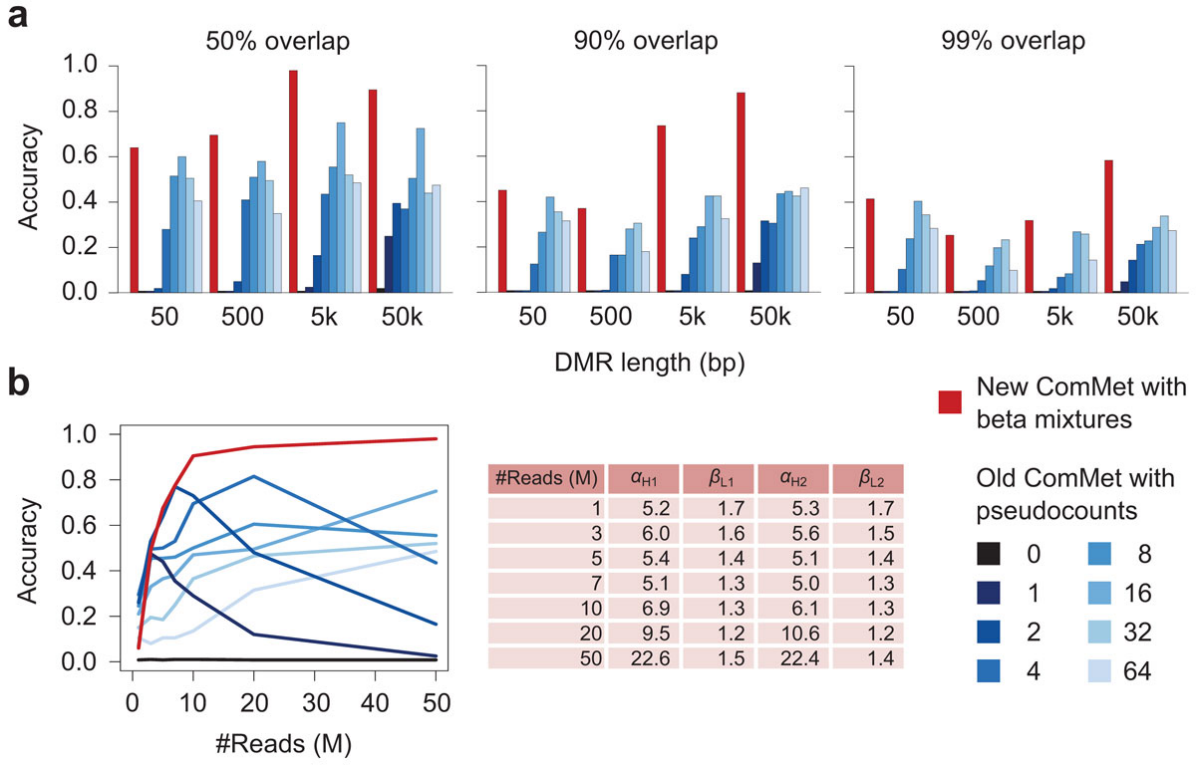
**Comparison between new and old ComMet evaluated on simulated data**. (a) For various DMR lengths and the fixed sequencing depth of 50M reads, accuracy evaluated with 50% (left), 90% (center), or 99% (right) reciprocal overlap is shown. (b) For varying sequencing depths and the fixed DMR length of 5000 bp, accuracy evaluated with 50% reciprocal overlap is shown. Also shown are estimated values of parameters in new emission functions.

**Figure 4:**
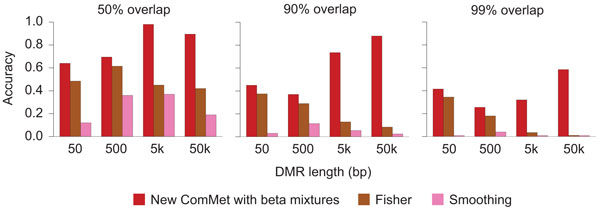
**Comparison between new ComMet and other methods evaluated on simulated data**. For various DMR lengths and the fixed sequencing depth of 50M reads, accuracy evaluated with 50% (left), 90% (center), or 99% (right) reciprocal overlap is shown.

Figures 3 and 4, and Figure S1 in Additional file 1 show the experimental results. ComMet using the new emission functions achieved better accuracy than that using the old ones with various values of the *pseudo *parameter (Figure 3a and Figure S1 in Additional file 1). It should be noted that, while the old emission functions attained comparable accuracy to the new ones when used with the optimal value of *pseudo*, it is difficult to find such optimal values in a practical situation where accuracy cannot be systematically evaluated. In fact, as explained in the "Methods" section, the optimal value of *pseudo *critically depends on sequencing depth, while the parameters in the new emission functions were successfully adjusted by our learning algorithms (Figure 3b and Figure S1 in Additional file 1). ComMet with the new emission functions also achieved better accuracy than Fisher's exact test and the smoothing method (Figure 4).

### Experiments on real data

We conducted experiments that evaluate agreement between detected DMRs and changes in gene expression. Note that similar experiments have been employed also in previous studies [6,11]. We collected data from [16], where human breast cancer and normal breast are measured by both RNA-seq and bisulfite-seq. We aligned RNA-seq reads to the human genome using TopHat [21]. Gene expression was measured by fragments per kilobase of transcript per million mapped reads (FPKM) using Cufflinks [22]. Differentially expressed genes (DEGs) were determined by the threshold of five-fold FPKM change. We evaluated agreement between DEGs and detected DMRs according to the previous study [6,11]. We focused on DEGs whose ±5 kbp regions around transcription start sites (TSSs) contained detected DMRs. The numbers of DEGs were counted for the top 1000 and 3000 DMRs detected by each method. We used these counts as a measure of the agreement. For the base-line of accuracy, we calculated the expected number of DEGs when DMRs were randomly placed in the TSS windows (denoted by *random guessing*).

In addition, we evaluated agreement between detected DMRs and changes in DNase I hypersensitivity as conducted in [6]. We collected data from [19], where human foreskin fibroblasts and embryonic stem cells are measured by bisulfite-seq. For these cell types, we obtained DNase I hypersensitivity data from the ENCODE project http://ftp.ebi.ac.uk/pub/databases/ensembl/encode/integration_data_jan2011/byDataType/openchrom/jan2011/fdrPeaks/. The data for each cell type contain the set of 150-bp regions that show the local maxima of DNase I hypersensitivity with false discovery rate (FDR) less than 1%. We defined "differentially sensitive sites" (DSSs) as those 150-bp regions present in either one of the two cell types. The agreement between DSSs and detected DMRs was evaluated similarly to the experiment for DEGs. We focused on DSSs whose ±5 kbp regions around the midpoints contained detected DMRs. The numbers of DSSs were counted for the top 1000 and 3000 DMRs detected by each method. We used these counts as a measure of the agreement.

**Figure 5:**
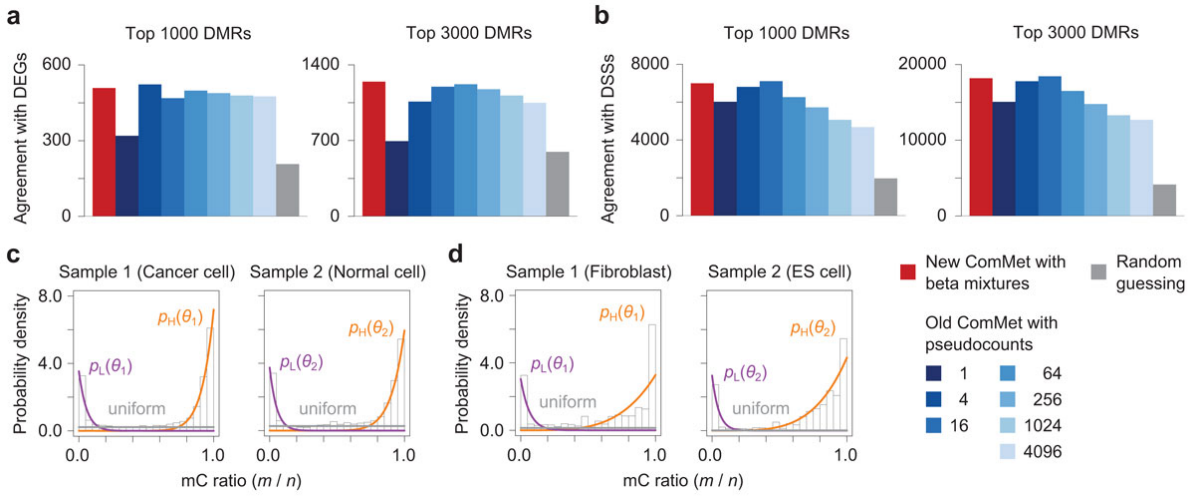
**Comparison between new and old ComMet evaluated on real data**. (a) Agreement between detected DMRs and changes in gene expression. (b) Agreement between detected DMRs and changes in DNase I hypersensitivity. See the main text for details. (c,d) The bimodal distributions of methylation levels observed in real data, and the fitted beta mixtures.

**Figure 6:**
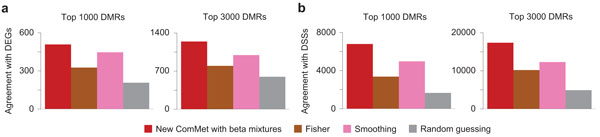
**Comparison between new ComMet and other methods evaluated on real data**. (a) Agreement between detected DMRs and changes in gene expression. (b) Agreement between detected DMRs and changes in DNase I hypersensitivity. See the main text for details.

Figures 5 and 6 show the experimental results. The advantage of the new emission functions over the old ones was also validated on real data, achieving better agreement of detected DMRs with DEGs (Figure 5a) and DSSs (Figure 5b). We again emphasize that the performance of the old emission functions critically depends on the choice of *pseudo *values, while the optimal value is difficult to find empirically. In contrast, as shown in Figure 5cd, the bimodal distributions of methylation levels were observed in real datasets, and our learning algorithms successfully fitted the beta mixtures. The new ComMet achieved better accuracy than Fisher's exact test and the smoothing method also for real datasets (Figure 6).

## Conclusions

In this paper, we described the new emission functions for HMM-based DMR detection from bisulfite-seq data. We proposed to incorporate the information of genome-wide methylation level distributions into emission functions, replacing the empirical parameter used in our previous study. ComMet with the new emission functions successfully improved DMR detection accuracy compared to the previous version. Recent studies suggest that detection of DMCs at individual cytosine sites can be improved by considering genome-wide methylation level distributions [13-15]. Therefore, our results have shown that such information is useful not only for detecting DMCs, but also for DMR detection. Furthermore, our HMM-based method achieves better accuracy than other methods using Fisher's exact test and methylation level smoothing. The implementation of ComMet is distributed as a part of Bisulfighter package, which is available at http://epigenome.cbrc.jp/bisulfighter.

## List of abbreviations

DMR: differentially methylated region; DMC: differentially methylated cytosine; HMM: hidden Markov model; DEG: differentially expressed gene; DSS: differentially sensitive site.

## Competing interests

The authors declare that they have no competing interests.

## Authors' contributions

YS developed the method, wrote the program, performed the experiments, and drafted the manuscript. TM conceived of the study, and coordinated the project. All authors have read and approved the final manuscript.

## Supplementary Material

Additional file 1 **Figure S1**. Benchmark for DMR detection at varying sequencing depth. For each DMR length, accuracy evaluated with 50% reciprocal overlap is shown. Also shown are estimated values of parameters in new emission functions. Click here for file

## Declarations

The publication charges for this article have been funded by Core Research for Evolutional Science and Technology (CREST) from Japan Science and Technology Agency (JST).

This article has been published as part of *BMC Genomics *Volume 16 Supplement 12, 2015: Joint 26th Genome Informatics Workshop and 14th International Conference on Bioinformatics: Genomics. The full contents of the supplement are available online at http://www.biomedcentral.com/bmcgenomics/supplements/16/S12.
