# Supplementary material for: Detection of differentially methylated regions from bisulfite-seq data by hidden Markov models incorporating genome-wide methylation level distributions
Source: BMC Genomics. 2015 Dec 9;16(Suppl 12):S3. doi: 10.1186/1471-2164-16-S12-S3 (PMC4682380; doi:10.1186/1471-2164-16-S12-S3)
Supplement: Additional file 1 — Figure S1. Benchmark for DMR detection at varying sequencing depth. For each DMR length, accuracy evaluated with 50% reciprocal overlap is shown. Also shown are estimated values of parameters in new emission functions. [file 1471-2164-16-S12-S3-S1.pdf]

a

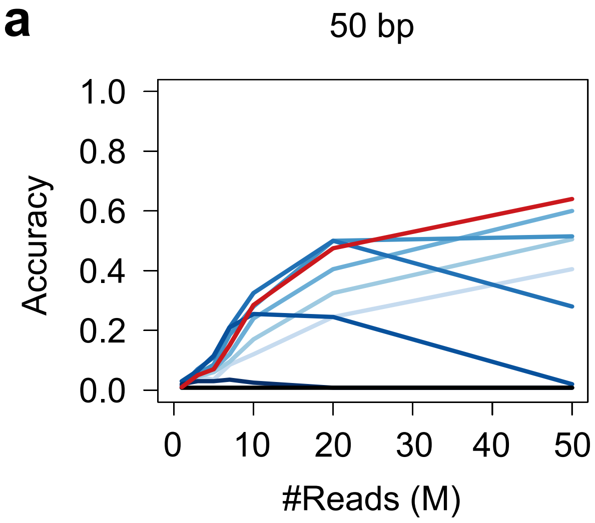

#Reads (M)	α_{H1}	β_{L1}	α_{H2}	β_{L2}
1	5.5	1.6	5.5	1.7
3	5.7	1.5	5.8	1.5
5	5.6	1.4	4.8	1.4
7	5.8	1.4	5.8	1.4
10	6.0	1.3	6.1	1.3
20	10.7	1.2	10.5	1.2
50	23.2	1.5	23.1	1.5

b

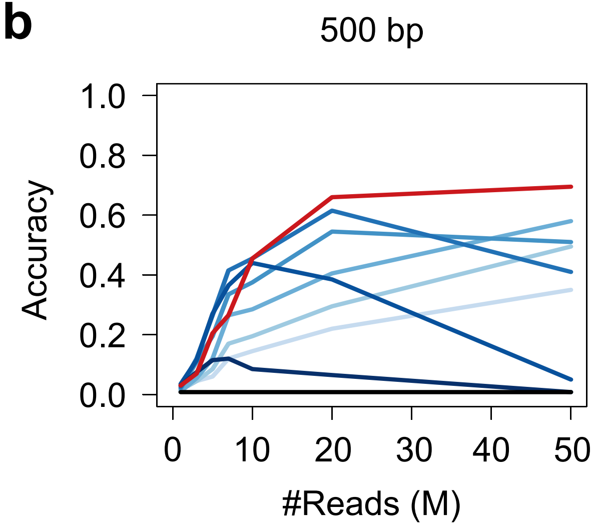

#Reads (M)	α_{H1}	β_{L1}	α_{H2}	β_{L2}
1	5.2	1.6	5.3	1.6
3	4.7	1.5	4.7	1.5
5	6.2	1.5	5.9	1.4
7	5.1	1.3	4.7	1.3
10	5.8	1.3	5.8	1.3
20	10.7	1.2	10.1	1.2
50	22.9	1.5	23.1	1.5

c

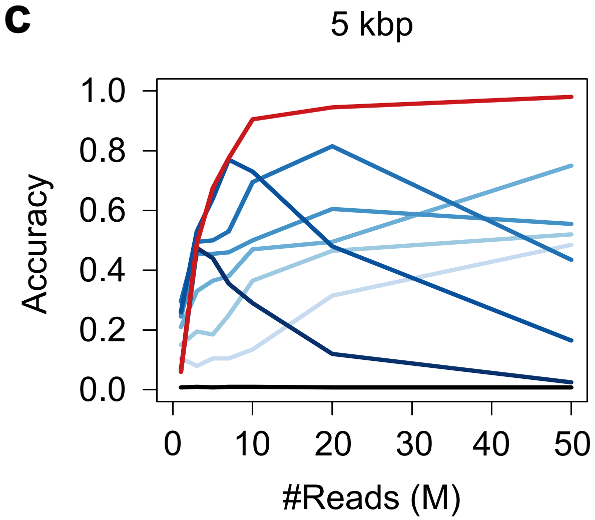

#Reads (M)	α_{H1}	β_{L1}	α_{H2}	β_{L2}
1	5.2	1.7	5.3	1.7
3	6.0	1.6	5.6	1.5
5	5.4	1.4	5.1	1.4
7	5.1	1.3	5.0	1.3
10	6.9	1.3	6.1	1.3
20	9.5	1.2	10.6	1.2
50	22.6	1.5	22.4	1.4

d

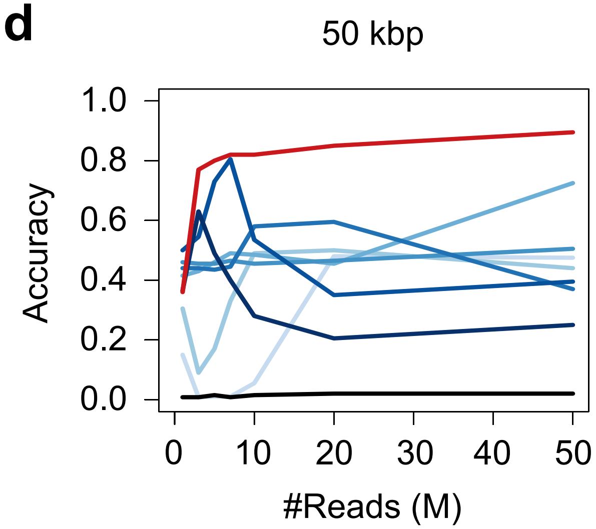

#Reads (M)	α_{H1}	β_{L1}	α_{H2}	β_{L2}
1	7.7	1.8	5.5	1.6
3	8.0	1.9	5.6	1.6
5	8.2	1.7	5.1	1.4
7	9.2	1.7	4.6	1.3
10	10.0	1.7	4.9	1.2
20	15.0	1.9	10.3	1.2
50	24.4	12.0	22.9	1.5

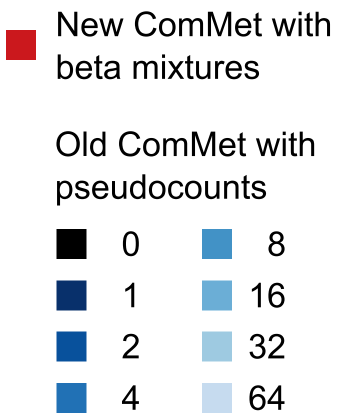

Figure S1. Benchmark for DMR detection at varying sequencing depth.

Benchmark for DMR detection at varying sequencing depth. For each DMR length, accuracy evaluated with 50% reciprocal overlap is shown. Also shown are estimated values of parameters in new emission functions.
